# Neoadjuvant and/or adjuvant immune checkpoint inhibitors combined with chemotherapy for locally advanced resectable penile squamous cell carcinoma

**DOI:** 10.3389/fimmu.2026.1731920

**Published:** 2026-03-19

**Authors:** Shanshan Xu, Feiran Chen, Lei Diao, Weiyu Wang, Xuemin Wang, Qiong Wu, Peipei Sun, Yating Hao, Yuqian Wang, Rongjie Ji, Yanan Jiang, Jun Du, Bing Ang, Zhigang Zhao, Qing Yang

**Affiliations:** 1 Department of Oncology, Tianjin Medical University Cancer Institute and Hospital, National Clinical Research Center for Cancer, Key Laboratory of Cancer Prevention and Therapy, Tianjin’s Clinical Research Center for Cancer, Tianjin, China; 2 Department of Medical Oncology, Tianjin First Central Hospital, School of Medicine, Nankai University, Tianjin, China; 3 Department of Urogenital Cancer, Tianjin Medical University Cancer Institute and Hospital, National Clinical Research Center for Cancer, Key Laboratory of Cancer Prevention and Therapy, Tianjin’s Clinical Research Center for Cancer, Tianjin, China

**Keywords:** advanced penile squamous-cell carcinoma, immune checkpoint inhibitors, neoadjuvant and/or adjuvant chemotherapy, neoadjuvant chemotherapy, programmed death-ligand 1

## Abstract

**Background:**

The efficacy of immune checkpoint inhibitors (ICI) combined with chemotherapy as a neoadjuvant/adjuvant therapy for locally advanced penile squamous-cell carcinoma (PSCC) remains unclear.

**Methods:**

A prospective, non-randomized, two-cohort study was designed to evaluate the efficacy of sintilimab combined with chemotherapy in this setting. Based on preoperative assessment, Patients were assigned to either undergo surgery following neoadjuvant therapy plus adjuvant therapy or to proceed directly to surgery followed by adjuvant therapy. Outcomes included progression-free survival (PFS), objective response rate (ORR), pathological complete response (pCR), major pathological response (MPR), overall survival (OS), disease control rate (DCR), and adverse events (AEs). The study has been registered with the Chinese Clinical Trial Registry (ChiCTR2400094629) and is currently ongoing.

**Results:**

A preliminary analysis was performed on 25 enrolled patients. With a median follow-up of 20 months, among the 17 patients who received neoadjuvant therapy, the ORR was 52.9% (including 2 complete responses and 7 partial responses), and the DCR was 76.5%. Notably, in the treatment-naive subgroup, the ORR reached 66.7%. Of the 12 patients who underwent surgery, 2 achieved pCR and 3 achieved MPR. The 18-month PFS and OS rates for the entire cohort were 71.4% and 90.0%, respectively. Stratified by treatment modality, the neoadjuvant-adjuvant group showed an 18-month PFS rate of 68.8% and an OS rate of 85.7%, while the adjuvant-only group had 18-month PFS and OS rates of 75.0% and 100.0%, respectively. Patients who attained a radiological CR/PR after neoadjuvant therapy had significantly better PFS compared to those with SD/PD (*P* = 0.005). No significant association was observed between PD-L1 expression (CPS ≥20) and PFS. Grade ≥3 treatment-related adverse events occurred in 20% of patients.

**Conclusion:**

This preliminary analysis demonstrates that ICI combined with chemotherapy provides durable antitumor activity and a manageable safety profile as neoadjuvant and/or adjuvant therapy for locally advanced PSCC. These results warrant further validation in larger cohorts.

## 1 Introduction

Being a rare disease, approximately 26000 new cases of squamous cell carcinoma of the penis (PSCC) are reported globally every year, mostly in developing countries (1, 2). However, the most important prognostic indicator in determining the long-term survival of PSCC patients is regional lymph node metastasis (3). Previous studies have reported the 5-year survival rates of >85% for patients with negative lymph nodes and 29%-40% for patients with positive lymph nodes (4, 5). Therefore, new therapeutic interventions are crucial to improving the survival rate of patients with locally advanced PSCC.

Neoadjuvant chemotherapy improves the disease response and long-term survival rates in patients with advanced inguinal and pelvic lymph node metastasis of penile cancer (6–10). Pagliaro et al. established the initial efficacy benchmark for the paclitaxel, ifosfamide, and cisplatin (TIP) regimen and demonstrated an objective response rate (ORR) of 50% and a pathological complete response (pCR) rate of 13.6% (7). Similarly, a retrospective analysis by Dickstein et al. reinforced that a response to chemotherapy can effectively predict survival outcomes (9). Another meta-analysis by Azizi et al. reported a pooled ORR of 53% and pCR of 16% for platinum-based regimens, suggesting a potentially more favorable toxicity profile for non-taxane-containing combinations (10). Additionally, postoperative radiotherapy and radiochemotherapy improve the disease control rate (DCR) and survival of locally advanced PSCC patients to varying degrees (3, 11, 12). Because of the disease’s rarity, it is challenging to conduct prospective randomized trials to determine the best therapeutic regimen for PSCC patients with inguinal lymph node metastasis or pelvic lymph node involvement.

A combination therapy based on immune checkpoint inhibitors (ICIs), including programmed death receptor ligand-1 (PD-L1) and programmed death receptor-1 (PD-1) inhibitors, has demonstrated prolonged survival benefits in several squamous cell carcinomas (13–15). Moreover, previous studies have demonstrated enhanced PD-L1 expression and dense infiltration of CD8+ T cells in the penile cancer microenvironment, thereby justifying the use of ICI combination therapies (16) Additionally, the ORR of ICI alone was 13% (n=11/85) in the overall cohort (locally advanced or metastatic) and 35% (n= 7/20) in patients with lymph node–only metastases in a study predominantly involving pretreated patients, with 80% having received ≥ second-line therapy (2). Yan et al. demonstrated that platinum-based chemotherapy combined with Anti-PD-1 antibody and epidermal growth factor receptor inhibition showed promising anti-tumor activity and exhibited 62.9% as well as 68.4% of 2-year overall survival (OS) rates and 2-year progression-free survival (PFS) rates in stage IV PSCC patients, respectively (17). Clinically, ICIs are also effective in a subset of PSCC patients (2, 18, 19). Notably, recent phase II trials have evaluated ICI and chemotherapy combinations as the first-line treatment for advanced PSCC. Although the HERCULES trial reported an ORR of 39.4% with pembrolizumab plus platinum-based chemotherapy (20), the EPIC-A trial suggested an ORR of 51.7% with cemiplimab and chemotherapy (21). These response rates represent a significant improvement over historical outcomes with chemotherapy alone, which yielded an ORR of 38.5% in a prior phase II study on metastatic PSCC patients undergoing a TPF (paclitaxel, cisplatin, and 5-fluorouracil) regimen (22).Together, these findings emphasize the importance of incorporating immunotherapy into the first-line treatment paradigm for advanced PSCC. However, the rationale of using this strategy at earlier disease stages, where the treatment intent shifts from palliation to cure, remains unclear. Direct extrapolation of outcomes from trials undertaken in the metastatic setting is challenging because of the distinct biological context and clinical goals in locally advanced, resectable PSCC.

Therefore, it is critical to evaluate the utility of ICI-based combination therapies early in the disease course, such as in neoadjuvant or adjuvant settings. Therefore, we initiated this prospective, phase II trial (ChiCTR2400094629) to investigate the efficacy and safety of sintilimab with chemotherapy as a neoadjuvant and/or adjuvant therapy for patients with locally advanced PSCC.

## 2 Patients and methods

### 2.1 Study design and patient selection

This prospective, non-randomized, two-cohort study was designed to evaluate the efficacy and safety of sintilimab plus chemotherapy as perioperative therapy for locally advanced PSCC, with a planned enrollment of 28 patients. Key inclusion criteria were: histologically confirmed PSCC with a large or inoperable primary tumors (T4 stage), palpable nodes ≥4 cm in diameter or fixed nodes, suspected extranidal extension or pelvic node involvement (N2/N3 stage); no evidence of distant metastasis; an Eastern Cooperative Oncology Group (ECOG) performance status of 0–1; and an age of ≥18 years. Major exclusion criteria included: prior treatment with a PD-1 or PD-L1 inhibitor, an active autoimmune disorder requiring systemic steroid therapy; and unstable systemic concomitant disease.

### 2.2 Treatment protocol and patient cohort

This prospective, non-randomized, two-cohort study was conducted at two centers (Tianjin First Central Hospital and Tianjin Medical University Cancer Institute & Hospital). Between May 6, 2022 and December 1, 2024, 26 patients were enrolled. This report presents a preliminary analysis of this cohort. Based on predefined resectability criteria outlined in the study protocol, enrolled patients were assigned to one of two therapeutic pathways following preoperative assessment. Assignment was determined by clinical evaluation and imaging studies (e.g., contrast-enhanced CT) according to the following criteria: patients who met any of the following conditions were assigned to the neoadjuvant-adjuvant pathway: 1) a primary penile tumor ≥5 cm in diameter; 2) inguinal lymph node metastasis with high-risk features (e.g., fixed nodes, invasion of femoral vessels, or deemed unsuitable for complete resection); or 3) presence of pelvic lymph node metastasis (N3). Patients who did not meet any of the above criteria were assigned to the adjuvant-only pathway, undergoing immediate radical surgery followed by adjuvant chemoimmunotherapy. The neoadjuvant-adjuvant pathway received 3-4 cycles of systemic therapy before planned surgery. The treatment regimen consisted of sintilimab (200 mg intravenously every 3 weeks) in combination with TP (nab-paclitaxel 260 mg/m²on day 1 plus cisplatin 25 mg/m² on days 1-3), administered in 21-day cycles. All patients were scheduled to complete 6 cycles of perioperative therapy. Eligible patients (including those in the adjuvant-only group and those achieving response or stable disease after neoadjuvant treatment) were to receive maintenance sintilimab until disease progression or for up to one year. The study protocol received approval from the Institutional Review Board, and written informed consent was obtained from all participants prior to enrollment.

### 2.3 Assessments

The primary endpoint was PFS based on the intention-to-treat population, defined as the time from treatment initiation to the first documented disease progression (per RECIST v1.1, confirmed by an independent review committee or investigator) or death from any cause, whichever occurred first. Secondary endpoints included ORR, DCR, major pathological response (MPR), pCR, OS, and adverse events (AEs). Tumor responses were assessed by contrast-enhanced computed tomography or positron emission tomography-computed tomography according to Response Evaluation Criteria in Solid Tumors version 1.1(RECIST v1.1), and classified as complete response (CR), partial response (PR), stable disease (SD), or progressive disease (PD). ORR was defined as the proportion of patients with an investigator-confirmed CR or PR (≥4 weeks after initial response). Upon blinded histopathological review of resected specimens, pCR was defined as the absence of residual viable tumor cells, and MPR was defined as the presence of ≤10% residual viable tumor cells. OS was defined as the time from treatment initiation to death from any cause. The disease control rate (DCR) was defined as the proportion of patients with a investigator-confirmed CR, PR, or SD (≥4 weeks after initial response). AEs were prospectively collected and graded according to the National Cancer Institute Common Terminology Criteria for Adverse Events version 5.0 (CTCAE V5.0). For safety analysis, all adverse events were retrospectively reviewed by investigators based on clinical presentation, timing, and potential association with immunotherapy to determine whether they were immune-related adverse events (irAEs). Exploratory analyses included the assessment of potential biomarkers, such as p16 and PD-L1 expression levels, which were assessed centrally for patients who provided sufficient baseline tumor tissue and research consent. PD-L1 expression was evaluated using the Combined Positive Score (CPS), calculated as (the number of PD-L1–staining cells [tumor cells, lymphocytes, macrophages]/the total number of viable tumor cells) × 100. A CPS of ≥20 was prespecified as the cutoff to define PD-L1 positivity (15).

### 2.4 Statistical analysis

This was a preliminary, exploratory phase II trial. The target enrollment of 28 patients was based primarily on clinical feasibility, reflecting the estimated number of eligible patients that could be recruited at the study centers within a reasonable timeframe. This sample size is consistent with the scale of prior clinical studies in PSCC (6–8, 23).

Patient, disease, and outcome characteristics were described using descriptive statistics. Short-term efficacy endpoints, including ORR and DCR, were calculated along with an exact 95% confidence interval (CI) using the exact Clopper-Pearson method. Time-to-event outcomes were estimated through the Kaplan-Meier method, with subgroup comparisons performed by the log-rank test. For PFS analysis, patients without an event at the data cutoff were censored at their last tumor assessment; for OS, surviving patients were censored at their last follow-up. Given the limited sample size, multivariable analysis was not conducted. A two-sided *P* value <0.05 was considered statistically significant. All statistical analyses were performed using SPSS version 27.0 (IBM, Armonk, NY, USA), and figures were generated with GraphPad Prism version 10.0 (GraphPad Software, San Diego, CA, USA).

## 3 Results

### 3.1 Baseline characteristics of patients

**Figure 1:**
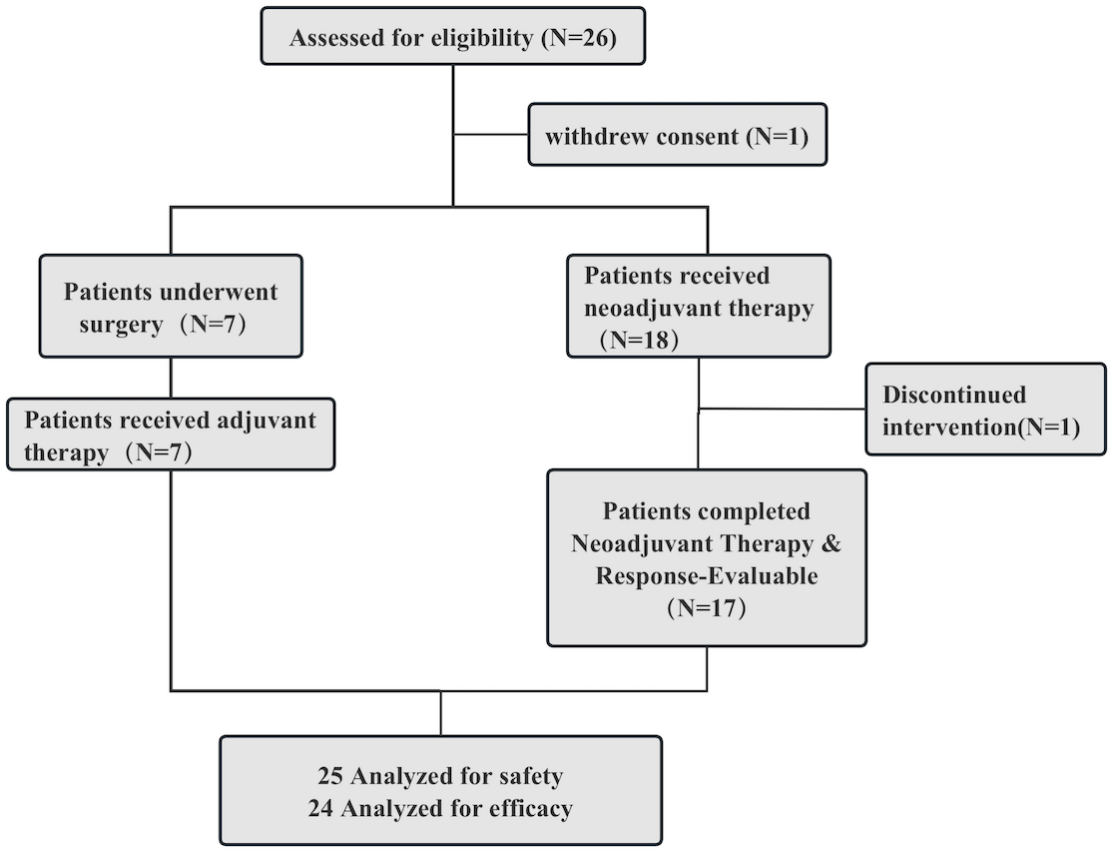
Flow diagram of patients with penile squamous cell carcinoma who underwent neoadjuvant and/or adjuvant therapy. Among 26 enrolled patients, one withdrew consent prior to treatment and another discontinued after the first cycle due to grade IV myelosuppression (excluded from efficacy analysis). Consequently, the safety and efficacy analysis sets included 25 and 24 patients, respectively.

A total of 26 patients with locally advanced PSCC were enrolled between May 6, 2022, and December 1, 2024, to receive treatment. One patient withdrew consent prior to any protocol treatment. Consequently, 25 patients comprised the safety and efficacy analysis populations. Following surgical assessment, 7 patients proceeded directly to surgery with adjuvant therapy, while the remaining 18 patients initiated neoadjuvant therapy. Of the latter, 17 were evaluable for treatment response; one patient discontinued treatment after the first cycle due to grade IV myelosuppression and was consequently excluded from the efficacy analysis but remained included in the safety analysis. An overview of the trial process is shown in **Figure 1**.

The median age was 55 years (interquartile range [IQR], 53-61), and all patients had an ECOG performance status scores of 0-1. All patients had lymph node metastasis, classified as N2 in 16 (64.0%) and N3 in 9 (36.0%). The majority of tumors (52.0%) were moderately differentiated (Grade 2) per the World Health Organization (WHO) grading system. Seventeen patients (68%) were treatment-naive, and 8 (32%) had received prior partial penectomy.

Biomarker analysis was performed on the 19 patients (76.0% of the cohort) with sufficient baseline tumor tissue. Among them, 9 patients (47.4%) showed PD-L1 CPS < 20, and 10 patients (52.7%) showed PD-L1≥ 20. Additionally, 13 patients (68.4%) had P16-positive tumors. The pretreatment median neutrophil-to-lymphocyte ratio (NLR) for the entire cohort was 2.7. Baseline and tumor characteristics are shown in **Table 1**.

**Table 1:** Clinical parameters of 25 patients with neoadjuvant and/or adjuvant ICI combined with chemotherapy.

Characteristic	No. of patients n (%)
Age	
Median, 55	
≥55	12(48.0)
< 55	13(52.0)
ECOG performance status	
0	12(1)
1	13(5)
T stage	
Tx	4(5)
T1	7(28.0)
T2	3(12.0)
T3	9(36.0)
T4	2(8.0)
N stage	
N2	16(64.0)
N3	9(36.0)
Disease Status	
Treatment-naive	17(68.0)
Pretreated	8(32.0)
Degree of differentiation	
Well differentiation	2(8.0)
Well- moderately differentiation	6(24.0)
Moderately differentiation	13(52.0)
Moderately- poorly differentiation	1(4.0)
Unknown	3(12.0)
NLR	
Median, 2.7	
> Median NLR	14(56.0)
< Median NLR	11(44.0)
PD-L1 IHC (n=19)	
CPS < 1	1(5.3)
CPS ≥ 1 < 20	8(42.1)
CPS ≥ 20 < 50	4(21.1)
CPS ≥ 50	6(31.6)
P16 IHC (n=19)	
Negative	6(31.6)
Positive	13(68.4)

### 3.2 Tumor and pathological response

Of the 17 patients evaluable for neoadjuvant therapy, the following responses were observed: CR in 2 (11.8%), PR in 7 (41.2%), SD in 4 (23.5%), and PD in 4 (23.5%). The ORR was 52.9% (95% CI: 31.0–73.8%), and the DCR was 76.5% (95% CI: 52.7-90.4%). The treatment-naive patient subgroup (N = 12) demonstrated a ORR of 66.7% (8/12), including 1 CR and 7 PR. Twelve of the 17 patients subsequently underwent radical surgery, and 2/12 (16.7%) achieved pCR, while 3/12 (25%) achieved MPR (**Table 2**). The remaining five patients did not proceed to surgery due to: disease progression (n=2, who transitioned to alternative systemic therapy); ineligibility for surgery (n=1, managed with systemic therapy); or patient refusal (n= 2, who opted for active surveillance).

**Table 2:** Treatment response and surgical outcomes to neoadjuvant immunochemotherapy, stratified by disease status.

Vairable	Overall patients (N = 17)	Naive patients (N = 12)	Recurrent patients (N = 5)
Clinical Response, n (%)			
CR	2 (11.8)	1(8.3)	1(20.0)
PR	7 (41.2)	7(58.3)	0(0.0)
SD	4 (23.5)	2(16.7)	2(40.0)
PD	4(23.5)	2(16.7)	2(40.0)
ORR, % (95%CI)	52.9 (31.0–73.8)	66.7 (34.9- 90.1)	20.0 (0.5- 71.6)
DCR, % (95%CI)	76.5 (52.7-90.4)	83.3 (51.6- 97.9)	60.0 (14.7- 94.7)
Surgical Outcomes, n			
Underwent Surgery	12	8	4
pCR	2/12	1/8	1/4
MPR	3/12	2/8	1/4
No Surgery	5	4^a^	1^b^

^a^ Surgery was not performed in four patients: one due to disease progression, one due to ineligibility, and two who declined surgery following partial response.

^b^ Surgery was not performed due to disease progression.

### 3.3 Survival outcomes

**Figure 2:**
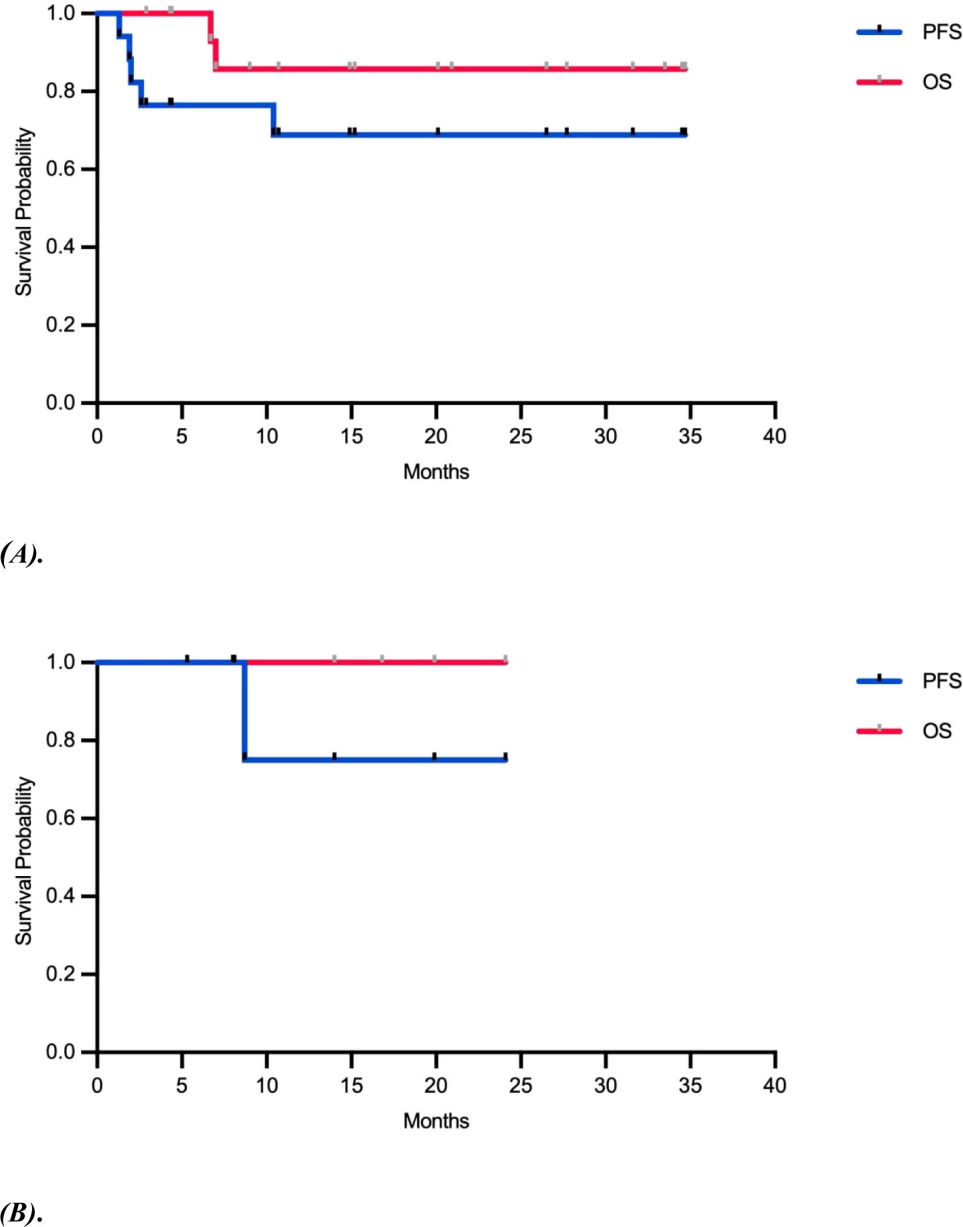
Kaplan-Meier survival curves of progression-free survival (PFS) and overall survival (OS), stratified by treatment arm. **(A)** PFS and OS for the neoadjuvant-adjuvant arm. **(B)** PFS and OS for the adjuvant-only arm.

As of May 2025, the median follow-up time was 20 months. The median PFS and OS were not reached. Outcomes stratified by treatment modality are shown in **Figure 2**. The neoadjuvant-adjuvant group had 6- and 12-month PFS rates of 76.4% and 68.8%, and OS rates of 100.0% and 85.7%, respectively (**Figure 2A**). The adjuvant-only group had PFS rates of 100.0% and 75.0%, with OS rates sustained at 100.0% at both time points (**Figure 2B**). The 18-month PFS and OS rates were consistent with the 12-month outcomes. For the entire cohort, the 6- and 12-month PFS rates were 83.3% and 71.4%, respectively, with corresponding OS rates of 100.0% and 90.0% (**Supplementary Figure 1**). The results are summarized in **Table 3**.

**Table 3:** Survival outcomes (irRECIST) according to follow-up.

Outcome	Overall cohort (N = 24)	Neoadjuvant-adjuvant group (N = 17)	Adjuvant group (N = 7)
PFS, % (95% CI)			
At 6 mo	83.3 (60.2–93.1)	76.4(48.0-89.3)	100.0 (100.0-100.0)
At 12 mo	71.4 (44.5–84.1)	68.8 (39.2-84.8)	75.0 (12.6-93.5)
At 18 mo	71.4 (44.5–84.1)	68.8 (39.2-84.8)	75.0 (12.6-93.5)
OS, % (95% CI)			
At 6 mo	100 (100.0–100.0)	100.0 (100.0-100.0)	100.0 (100.0-100.0)
At 12 mo	90.0 (64.9–96.5)	85.7 (52.7-95.1)	100.0 (100.0-100.0)
At 18 mo	90.0 (64.9–96.5)	85.7 (52.7-95.1)	100.0 (100.0-100.0)

Data for the treatment subgroups are presented for exploratory, descriptive purposes only. Due to the non-randomized assignment based on resectability criteria, these groups are not directly comparable.

### 3.4 Exploratory and subgroup analyses

**Figure 3:**
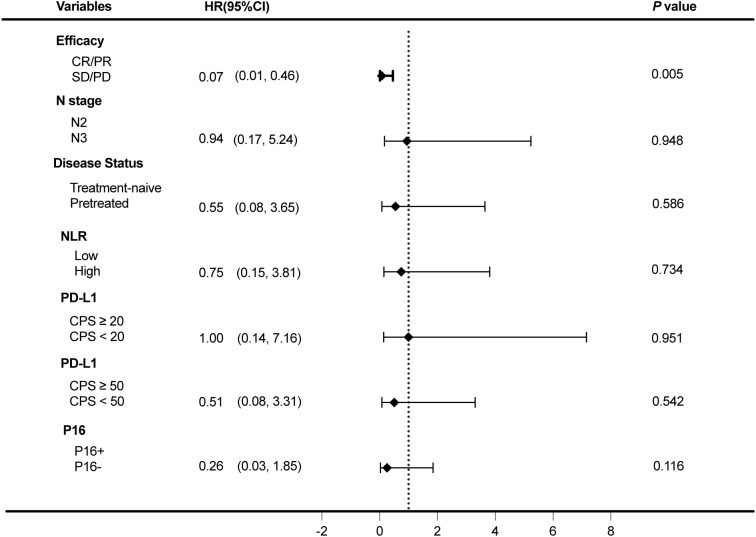
Forest plot of subgroup survival analyses. The plot displays outcomes for the following subgroups: efficacy in Neoadjuvant patients, N stage in patients, treatment-naive and recurrent patients, NLR, PD-L1 IHC status and P16 IHC status, *p*-values were calculated by Log-rank test.

Exploratory subgroup analyses of PFS are presented in **Figure 3**. Patients who achieved CR/PR with neoadjuvant therapy had significantly longer PFS than those who with SD/PD (*P* = 0.005, HR 0.07, 95% CI: 0.01-0.46). No statistically significant differences in PFS were observed between N2 and N3 patients (*P* = 0.948, HR 0.94, 95% CI: 0.17-5.24), treatment-naive and previously treated patients (*P* = 0.586, HR 0.55, 95% CI: 0.08-3.65), or low-and high-NLR groups (*P* = 0.734, HR 0.75, 95% CI: 0.15-3.81). PD-L1 expression (CPS ≥20 vs. <20) was not significantly associated with PFS (*P* = 0.951, HR 1.00, 95% CI: 0.14-7.16). A non-significant trend toward improved PFS was observed in patients with CPS ≥50 compared to those with CPS <50 (*P* = 0.542, HR 0.51, 95% CI: 0.08-3.31). and in patients with p16-positive tumors (*P* = 0.116, HR 0.26, 95% CI: 0.03-1.85).

### 3.5 Evaluation of adverse events

Grade 3-4 AEs occurred in 5 patients (20%), including anemia, neutropenia, arthralgia and rash, as detailed in **Table 4**. The most frequently reported adverse events of any grade were alopecia (n=13, 52.0%) and peripheral sensory neuropathy (n=12, 48.0%), irAEs of any grade included: decreased appetite (n=4, 16.0%), fatigue (n=1, 4.0%), rash (n=2, 8.0%), hypothyroidism (n=1, 4.0%), hyperglycemia (n=1, 4.0%), and increased aminotransferase (n=1, 4.0%). No treatment-related deaths were reported. By the data cutoff date, 6 patients had discontinued antitumor treatment, primarily due to PD (n=5), one patient discontinued due to grade IV myelosuppression.

**Table 4:** Main adverse events with treatment in patients.

Adverse events	AEs		irAEs	
	ALL grades, n (%)	Grade 3–4, n (%)	ALL grades, n (%)	Grade 3–4, n (%)
Alopecia	13(52.00)	–	–	–
Peripheral sensory neuropathy	12(48.00)	–	–	–
Decreased appetite	9(36.00)	–	4(16.00)	–
Fatigue	3 (12.00)	–	1 (4.00)	–
Arthralgia	3 (12.00)	1 (4.00)	–	–
Nausea	3 (12.00)	–	–	–
Neutropenia	2 (8.00)	2 (8.00)	–	–
Rash	2 (8.00)	1 (4.00)	2 (8.00)	1(4.00)
Anemia	1 (4.00)	1 (4.00)		–
Hypothyroidism	1 (4.00)		1 (4.00)	–
Hyperglycemia	1 (4.00)		1 (4.00)	
Increased aminotransferase	1 (4.00)	–	1 (4.00)	–

### 3.6 Individual treatment outcomes

**Figure 4:**
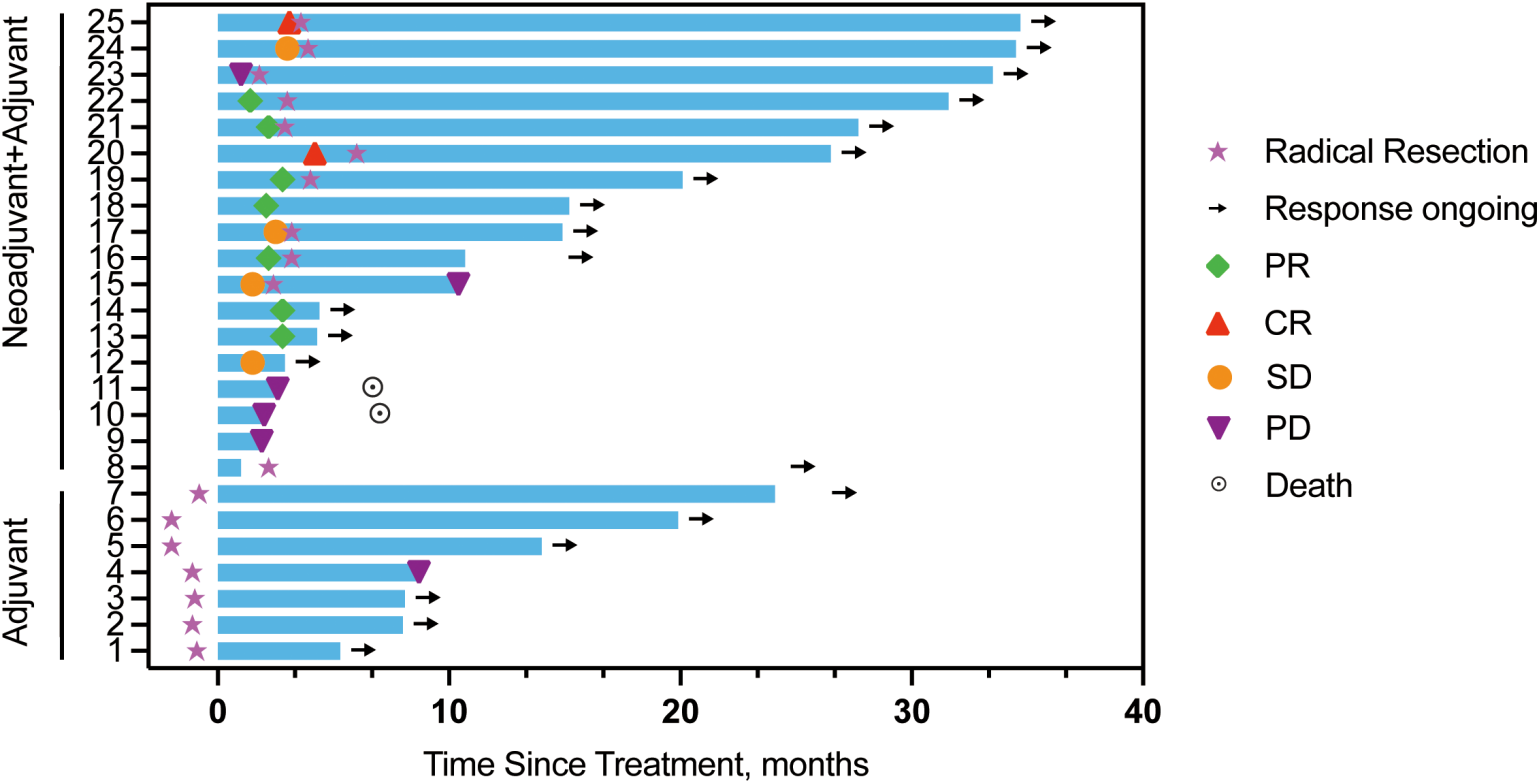
Time to and duration of response in the total cohort. Timelines of resection and disease assessment for each of the 25 participants in this study are shown. Individual patient treatment trajectories are annotated with corresponding identifiers (e.g., Patient #24).

Among the 25 patients, 20 (14 in the neoadjuvant -adjuvant group and 6 in the adjuvant-only group) showed no evidence of disease recurrence or progression, as detailed in **Figure 4**. The longest recorded PFS was 34.7 months, observed in a patient from the neoadjuvant-adjuvant group (Patient #25) who underwent partial penectomy and lymph node dissection.

One patient (Patient #18) achieved a 77% reduction in tumor burden after neoadjuvant therapy but declined surgery and further treatment, maintaining a PFS of 15.2 months at the last assessment. Two other patients who achieved PR did not undergo surgery: one (Patient #14) refused for personal reasons, and the other (Patient #13) was deemed surgically ineligible due to a high risk of positive margins.

Among patients who progressed with neoadjuvant therapy, one (Patient #23) had inguinal lymph node enlargement and subsequently underwent radical surgery with complete resection of the tumor. The patient remained recurrence-free and had a PFS of 33.5 months as of the data cutoff. Another patient (Patient #10) underwent partial penile resection and inguinal lymph node dissection, but unfortunately, the tumor had an incomplete resection. One month after surgery, inguinal lymph node enlargement and pelvic lymph node metastasis were observed. Subsequent treatment (toripalimab + niraparib + cisplatin + fluorouracil + ifosfamide) was ineffective, and the patient had an OS of 7.0 months. Patient #11 had PD after neoadjuvant therapy and failed second-line treatment, with an OS of 6.7 months.

In the adjuvant-only group (n=7), one (Patient #7) completed treatment and was under observation with the longest PFS in this subgroup (26 months). Five patients (Patient #1, #2, #3, #5, #6) were still receiving adjuvant therapy without recurrence. One (Patient #4), who experienced disease progression (soft tissue metastasis in the inguinal region) after four cycles of sintilimab maintenance therapy, initiated second-line chemotherapy.

## 4 Discussion

Despite evolving therapeutic strategies, the prognosis of stage N2 or N3 (stage III or stage IV) PSCC patients remains poor (4, 5). Hence, neoadjuvant or adjuvant chemotherapy are currently being used in cases with locally invasive or fixed inguinal lymph nodes (6, 24). However, the research on neoadjuvant and/or adjuvant treatment strategies after node-positive inguinal lymphadenectomy is relatively scarce. A non−prospective study on 32 patients with locally advanced or metastatic PSCC, in which all of them received atezolizumab (combined with radiotherapy in 20 patients), showed that patients achieved a median progression−free survival of 5.3 months, suggesting the clinical efficacy of ICIs alone or with radiotherapy in this population (19). Additionally, ICIs with chemotherapy as a neoadjuvant or in an adjuvant role may provide clinical benefits for patients with locally advanced squamous cell carcinoma, including head and neck squamous cell carcinoma (25, 26), esophageal squamous cell carcinoma (13), and lung squamous cell carcinoma (27, 28). Hence, we sought to explore the efficacy of ICIs with chemotherapy as an approach for treating locally advanced PSCC cases. To our knowledge, our trial is the first prospective study to investigate the efficacy of the ICI and TP (paclitaxel plus cisplatin) regimen as a neoadjuvant and/or adjuvant strategy for PSCC.

In our study, 71.4% of the enrolled stage N2 or N3 PSCC patients remained free of progression at 18 months after their last clinical assessment. The regimen yielded an ORR of 52.9% for the neoadjuvant arm; a numerically higher ORR of 66.7% was observed in treatment-naive patients. Furthermore, a superior PFS was observed in PSCC patients with pCR and MPR post-neoadjuvant therapy. Notably, this efficacy was achieved with a manageable safety profile, which was defined by a reduced incidence (20.0%) of grade 3-4 adverse events. Thus, this study adds a new dimension to the immunotherapy landscape for PSCC. Although the recent retrospective analysis by Schieber et al. in metastatic PSCC has demonstrated the use of ICIs in advanced disease, this study provides the initial evidence for the feasibility and preliminary efficacy of a perioperative chemoimmunotherapy regimen in locally advanced, potentially resectable PSCC (29).

This study was predicated on the recommendation for neoadjuvant and adjuvant chemotherapy to treat PSCC patients with bulky inguinal lymphadenopathy or unresectable primary tumors, although there is no evidence regarding its benefits from randomized trials (24). Neoadjuvant TIP chemotherapy induces clinically effective responses in PSCC patients with regional lymph node metastases (6, 7). However, the survival rate of the TIP neoadjuvant therapy should be improved further, and the significant toxic adverse effects have affected the application of the TIP regimen (30). In this study, we evaluated the ICI combined TP regimen to explore a novel neoadjuvant and/or adjuvant therapy that might be less toxic and more efficient. The TP regimen is effective for squamous cell carcinoma; its side effects are significantly lower than those of the TIP regimen (30). Furthermore, a retrospective study demonstrated first-line treatment with a PD-1 inhibitor plus TP chemotherapy was effective and tolerable in advanced PSCC, yielding a median progression-free survival of 15.0 months (95% CI, 11.4 to not reached) (23).In addition, the combination of PD-1 and TP as a neoadjuvant therapy significantly improves the pCR and MPR rates in patients with locally advanced head and neck squamous cell carcinoma when compared to the traditional TP regimen (26). These findings, along with the usage of neoadjuvant ICI in lung squamous cell carcinoma and esophageal squamous cell carcinoma, provide rationale for evaluating the utility of neoadjuvant and/or immune checkpoint inhibition in PSCC. The ICI employed in this trial, sintilimab, is a completely human anti-PD-1 IgG4 monoclonal antibody. Thus, using sintilimab with chemotherapy augments antitumor immunity by capitalizing on the chemotherapy’s immunomodulatory effects (31). This regimen has shown a promising clinical activity and a favorable cost-effectiveness profile in several malignancies, notably achieving a pathological complete response rate of 32.6% as neoadjuvant therapy for locally advanced cervical squamous cancer patients (32).

In our neoadjuvant arm, the ORR of the ICI and TP regimen was 52.9%, including 2 (11.7%) and 7 (41.1%) cases of CR and PR, respectively. However, the superior ORR observed in treatment-naive patients (66.7%), compared to the overall cohort (52.9%), underscores the disease status as a predictor of efficacy. From a biological perspective, it is suggested that prior therapies can foster an enhanced immunosuppressive tumor microenvironment. Consequently, treatment-naive patients are more likely to possess a competent immune system, with T-cells that have not undergone the exhaustive processes induced by previous treatment pressures, thereby enabling a more robust response to initial immunochemotherapy (33). This rationale is consistent with significant outcomes of first-line immunotherapy in other malignancies, like non-small cell lung cancer and melanoma (34, 35). Therefore, our data highlight that treatment-naive individuals demonstrated a promising ORR (66.7%) to neoadjuvant immunochemotherapy in this cohort. Twelve cases (70.5%) underwent radical penectomy post-neoadjuvant therapy. The 18-month PFS rates were 68.8% in patients (n=17) on neoadjuvant-adjuvant therapy. Among our seven patients receiving adjuvant therapy, their 18-month OS rates were 100.0% (n=7); only one case developed PD after four maintenance treatment cycles, and the other six cases exhibited no recurrence or metastasis at the time of data analysis. Furthermore, with the median observation period of 20 months, there were only two deaths reported among all patients who received therapy, with a maximum survival of 34.7 months. However, the fact that median PFS and OS were not reached at a median follow-up of 20 months is a significant finding. Hence, the reported 18-month survival rates should be viewed as early milestone metrics, with mature survival outcomes awaiting a longer follow-up. Our results demonstrate that the combined ICI and TP regimen was well tolerated, with zero deaths attributed to chemotherapy-related toxicity. Although most patients reported mild adverse events, only 20.0% of the AEs were grade ≥3, and only one patient experienced a grade ≥3 irAEs. The promising response, survival outcomes and favorable safety profile observed in our study can help other randomized controlled trials in evaluating the efficacy of immunotherapy in PSCC.

Human papillomavirus (HPV) infection is an important carcinogenic factor in PSCC (36, 37). Although HPV infection is correlated with a good prognosis post-ICI treatment in head and neck squamous cell carcinoma (38), its role as a prognostic factor in PSCC remains unclear. Vries et al. demonstrated that superior PFS was observed in HPV-positive advanced PSCC patients who received ICI and radiotherapy (19). Wei et al. also demonstrated that HPV infection might confer survival benefits in advanced PSCC patients who are receiving first-line ICI+chemotherapy+anti-Epidermal Growth Factor Receptor (37). However, no association was observed between the HPV infection and PFS in our cohort. The reason why our results are inconsistent with other studies may be due to the differences in disease staging and therapeutic regimes among the enrolled patients. However, many studies have also found no difference between HPV-negative and HPV-positive patients regarding nodal metastasis or survival in the era of chemoradiotherapy (39–41).

An important prognostic biomarker, PD-L1 expression, helps to select patients for ICI treatment (15). Among 19 cases in our study with PD-L1 data, 18 samples exhibited ≥1 CPS, and 10/19 (52.6%) had ≥20 CPS. In a previous study, PD-L1-high tumors (CPS ≥20) exhibited better PFS compared with PD-L1-low tumor patients in advanced head and neck squamous cell carcinoma cases (15). However, we did not observe any association between PD-L1 expression and PFS in PSCC patients. This finding is consistent with other studies that did not demonstrate differences in survival with PD-L1 expression status (42, 43).

Undergoing penectomy can permanently affect sexual health and psychological well-being in PSCC patients (44, 45). The implications for quality of life are significant. In our study, one patient experienced a 77% reduction in the lesion’s size post-neoadjuvant therapy. The patient refused surgery and entered surveillance. The PFS is now 15.2 months. Additionally, two patients who were initially diagnosed with metastases achieved complete remission after being treated with a combination of ICI and chemotherapy. They are also currently undergoing continuous CR and surveillance. Cercek et al. demonstrated that ICI alone is significantly effective in mismatch repair-deficient, locally advanced rectal cancer. All 12 participants achieved complete remission and did not undergo surgery (46). Although we did not evaluate this specific molecular marker, our significant results after immunotherapy in PSCC support a parallel investigative premise. Thus, an immunotherapy-based organ-preservation strategy warrants dedicated exploration in PSCC.

Limitations of this study include its two-cohort, non-randomized design, which introduced selection bias, as evidenced by the higher baseline tumor burden in patients receiving neoadjuvant therapy—a reflection of real-world clinical triage rather than randomization. The small sample size restricts interpretation, and although clinical response correlated with prolonged progression-free survival (PFS), the specific contribution of immunotherapy remains uncertain. Subgroup analyses (e.g., PD-L1, p16) are exploratory and require further validation in larger cohorts. Larger, multi-center studies with stratified analyses based on prior treatment exposure are warranted to validate these findings.

In conclusion, our prospective, non-randomized, two-cohort, exploratory study provides preliminary evidence to support the antitumor activity and safety profile of ICI-chemotherapy combination in high-risk PSCC, thereby justifying its evaluation in future controlled trials.

## Data Availability

The raw data supporting the conclusions of this article will be made available by the authors, without undue reservation.
