# Correction: Neoadjuvant and/or adjuvant immune checkpoint inhibitors combined with chemotherapy for locally advanced resectable penile squamous cell carcinoma

**DOI:** 10.3389/fimmu.2026.1863014

**Published:** 2026-04-30

**Authors:** Shanshan Xu, Feiran Chen, Lei Diao, Weiyu. Wang, Xuemin Wang, Qiong Wu, Peipei Sun, Yating Hao, Yuqian Wang, Rongjie Ji, Yanan Jiang, Jun Du, Bing Ang, Zhigang Zhao, Qing Yang

**Affiliations:** 1 Department of Oncology, Tianjin Medical University Cancer Institute and Hospital, National Clinical Research Center for Cancer, Key Laboratory of Cancer Prevention and Therapy, Tianjin’s Clinical Research Center for Cancer, Tianjin, China; 2 Department of Medical Oncology, Tianjin First Central Hospital, School of Medicine, Nankai University, Tianjin, China; 3 Department of Urogenital Cancer, Tianjin Medical University Cancer Institute and Hospital, National Clinical Research Center for Cancer, Key Laboratory of Cancer Prevention and Therapy, Tianjin’s Clinical Research Center for Cancer, Tianjin, China

**Keywords:** advanced penile squamous-cell carcinoma, immune checkpoint inhibitors, neoadjuvant and/or adjuvant chemotherapy, neoadjuvant chemotherapy, programmed death-ligand 1

The order of authors in the author list of the published paper was erroneously given as:

“Shanshan. Xu1,2†, Feiran. Chen3†, Lei Diao3, Weiyu. Wang3, Xuemin. Wang2, Qiong Wu2, Peipei. Sun2, Yating. Hao2, Yuqian. Wang2, Rongjie. Ji2, Yanan Jiang2, Jun Du3, Bing Ang2*, Qing Yang3*, Zhigang. Zhao2*”

The correct author list reads:

“Shanshan. Xu1,2†, Feiran. Chen3†, Lei Diao3, Weiyu. Wang3, Xuemin. Wang2, Qiong Wu2, Peipei. Sun2, Yating. Hao2, Yuqian. Wang2, Rongjie. Ji2, Yanan Jiang2, Jun Du3, Bing Ang2*, Zhigang. Zhao2*, Qing Yang3*”

The original version of this article has been updated.

Affiliation “3Department of Urogenital Cancer, Tianjin Medical University Cancer Institute and Hospital, National Clinical Research Center for Cancer, Key Laboratory of Cancer Prevention and Therapy, Tianjin’s Clinical Research Center for Cancer, Tianjin, 300060, China.” was erroneously given as “3Department of Urology, Tianjin Medical University Cancer Institute and Hospital, National Clinical Research Center for Cancer, Key Laboratory of Cancer Prevention and Therapy, Tianjin’s Clinical Research Center for Cancer, Tianjin, 300060, China”.

The original version of this article has been updated.

