# Supplementary material for: Neoadjuvant and/or adjuvant immune checkpoint inhibitors combined with chemotherapy for locally advanced resectable penile squamous cell carcinoma
Source: Front Immunol. 2026 Mar 19;17:1731920. doi: 10.3389/fimmu.2026.1731920 (PMC13044089; doi:10.3389/fimmu.2026.1731920)
Supplement: Supplementary file 1 [file DataSheet1.pdf]

frontiers

Supplementary Figure

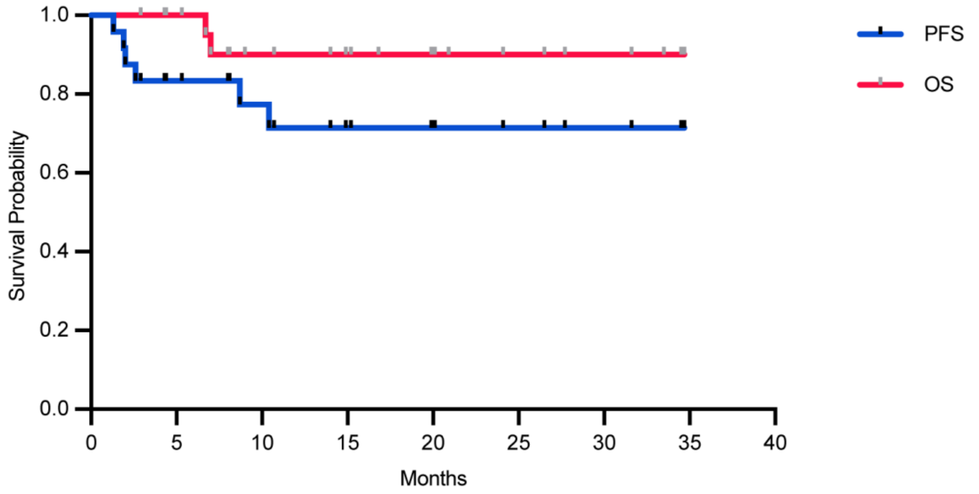

Supplementary Figure 1. Kaplan-Meier survival curves for the entire cohort.
